# Association between single nucleotide polymorphisms, *TGF-β1* promoter methylation, and polycystic ovary syndrome

**DOI:** 10.1186/s12884-023-06210-3

**Published:** 2024-01-02

**Authors:** Mengge Gao, Xiaohua Liu, Heng Gu, Hang Xu, Wenyao Zhong, Xiangcai Wei, Xingming Zhong

**Affiliations:** 1 NHC Key Laboratory of Male Reproduction and Genetics, Guangdong Provincial Reproductive Science Institute (Guangdong Provincial Fertility Hospital), Guangzhou, China; 2 Department of Public Health and Preventive Medicine, School of Medicine, Jinan University, Guangzhou, Guangdong 510630 China; 3 Guangdong Women and Children Hospital, Guangzhou, China; 4 Department of Clinical Nutrition, Huadu District People’s Hospital, 48 Xinhua Road, Huadu, Guangzhou, Guangdong 510800 China

**Keywords:** Polycystic ovary syndrome, Single nucleotide polymorphism, Transforming growth factor β1, TGF-β1, DNA methylation, PCOS epigenetics, Insulin resistance, Whole exome sequencing

## Abstract

**Background:**

Polycystic ovarian syndrome (PCOS) is a common endocrine and metabolic disease in women. Hyperandrogenaemia (HA) and insulin resistance (IR) are the basic pathophysiological characteristics of PCOS. The aetiology of PCOS has not been fully identified and is generally believed to be related to the combined effects of genetic, metabolic, internal, and external factors. Current studies have not screened for PCOS susceptibility genes in a large population. Here, we aimed to study the effect of TGF-β1 methylation on the clinical PCOS phenotype.

**Methods:**

In this study, three generations of family members with PCOS with IR as the main characteristic were selected as research subjects. Through whole exome sequencing and bioinformatic analysis, *TGF-β1* was screened as the PCOS susceptibility gene in this family. The epigenetic DNA methylation level of *TGF-β1* in peripheral blood was detected by heavy sulfite sequencing in patients with PCOS clinically characterised by IR, and the correlation between the DNA methylation level of the *TGF-β1* gene and IR was analysed. We explored whether the degree of methylation of this gene affects IR and whether it participates in the occurrence and development of PCOS.

**Results:**

The results of this study suggest that the hypomethylation of the CpG4 and CpG7 sites in the *TGF-β1* gene promoter may be involved in the pathogenesis of PCOS IR by affecting the expression of the *TGF-β1* gene.

**Conclusions:**

This study provides new insights into the aetiology and pathogenesis of PCOS.

**Supplementary Information:**

The online version contains supplementary material available at 10.1186/s12884-023-06210-3.

## Introduction

Polycystic ovary syndrome (PCOS) is a reproductive, endocrine, and metabolic disease with high clinical heterogeneity. It frequently occurs in women of reproductive age and is mainly characterised by chronic anovulation (ovulation dysfunction or loss) and hyperandrogenaemia (HA). The clinical manifestations of PCOS include menstrual disorders, polycystic ovarian changes, infertility, hypertrichosis, and acne. Patients may also have obesity, dyslipidaemia, insulin resistance, or other metabolic abnormalities [1]. Environmental and genetic factors are involved in the occurrence of PCOS; however, the specific mechanism remains unclear. Several studies on PCOS populations and mouse models have shown that there is a certain genetic susceptibility to PCOS and that symptoms such as HA, IR, and obesity are very similar across generations [2–5]. The incidence of PCOS is often significantly higher in a family, indicating that genetic factors play a role in its occurrence. Studies on identical and fraternal twins have shown that PCOS is a complex disease involving multiple genes [6]. Individual genes, gene-gene interactions, and gene-environment interactions influence the occurrence of PCOS. Epigenetic research highlights the complexity of PCOS aetiology. Ning et al. [7] showed that the DNA methylation patterns of PCOS patients differed from those in the normal control population. Qu et al. [8] reported that differential CpG island methylation in PPARG1 and NCOR1 of granule cells leads to HA and the subsequent development of ovarian dysfunction.

Studies have found that transforming growth factor β1(TGF-β1) is involved in PCOS via ovarian fibrosis [9–14], androgen synthesis [15–17], ovulation disorder [14, 18–21], and insulin resistance [13, 22–25].

PCOS susceptibility genes vary greatly among different families, indicating a complex multigene genetic predisposition. Therefore, owing to the clinical heterogeneity of PCOS, finding common susceptibility genes in multiple races and families according to different clinical characteristics may be a direction for studying the genetic mechanism of PCOS.

Although many PCOS susceptibility genes have been identified using genomics, it remains difficult to explain the complexity of its aetiology and clinical manifestations. Epigenetics has attracted increasing attention as a link between environmental and genetic factors. DNA methylation was one of the earliest and most thoroughly studied epigenetic regulatory mechanisms and refers to a biochemical modification process in which a specific base on the DNA sequence stably binds to a methyl group through covalent bonds under the catalysis of DNA methyltransferase. CpG loci are unevenly distributed in the genome, and regions with a high frequency can become CpG islands, which are mainly located near the promoter of the gene and in the first exon region. The DNA methylation we usually study refers to the methylation of the 5th carbon atom on the cytosine of the CpG islands [26]. By regulating the expression of various cytokines, DNA methylation may promote inflammatory responses, steroid synthesis signal transduction, and the dysregulation of glucose and lipid metabolism, thereby affecting the occurrence of diseases.

Many differentially methylated gene loci were found in the peripheral and umbilical cord blood, ovary, endometrium, skeletal muscle, adipose tissue, and hypothalamus of PCOS patients [27]; the multifunctional pathways of these loci are highly correlated with different clinical features of PCOS. However, the function of these genes is unclear, there is a lack of recognised diagnostic criteria, the degree of variation of the study samples is large, and the repeatability of the experiment is low; therefore, it is not clear how they affect the pathogenesis of PCOS.

Here, we conducted peripheral blood total exon sequencing was conducted for a three-generation PCOS family to find the susceptibility gene, based on the genetic aetiology of PCOS. Then, based on the multifunctional characteristics of TGF-β1, epigenetic aetiology was used to explore the effect of TGF-β1 methylation on the clinical PCOS phenotype, hopefully providing a new scientific basis for studying the pathogenesis of PCOS.

## Materials and methods

### Research objects

#### Diagnostic criteria

1. PCOS: According to the Rotterdam (Netherlands) PCOS diagnostic criteria: a. Sparse or anovulation. B. Clinical or biochemical tests showing hyperandrogenemia. C. Transvaginal or rectal ultrasound suggests polycystic ovarian changes: ≥12 small follicles 2 to 9 mm in diameter on at least one ovary and/or ovarian volume > 10 ml. PCOS can be diagnosed if two of the three conditions are met and other diseases are excluded [28].
2. IR: Among them, HOMA-IR > 2.69 can be diagnosed as insulin resistance (IR) symptoms; HOMA-IR = Fasting Plasma Glucose (FPG, mmol/L) ×fasting insulin (FINS, µU/ml)/22.5.
3. hyperandrogenemia (HA): T > 1.67nmol/L was diagnosed as HA.

#### Study participants

**Fig. 1:**
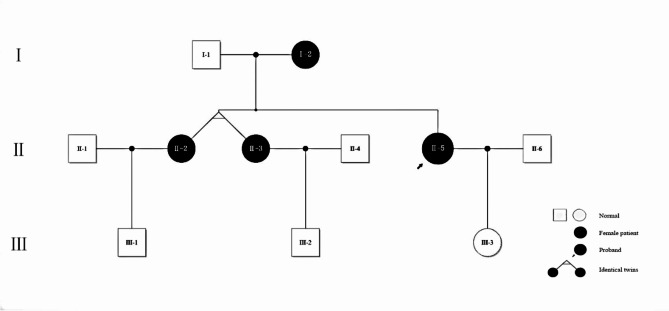
The PCOS family pedigree selected for this study. II-5 is the proband, II-2, II-3 are sisters of the proband, I-2 is the mother of II-2 and II-5, III-3 is the daughter of the proband

(1) PCOS family: This study included a PCOS Han family who visited the outpatient department of Guangdong Provincial Fertility Hospital in July 2020 without inbreeding (Fig. 1). Clinical data and blood samples from several female members of the family (I-2, II-2, II-5, III-3) were collected for genomic DNA isolation and genetic studies. I-2 is now menopausal, which can be inferred as PCOS according to previous menstruation. III − 3 is not menstruating now, so she cannot be clearly diagnosed as PCOS.

(2) Case group: PCOS patients treated in the outpatient department of Guangdong Provincial Fertility Hospital from 2020 to 2022 were included as case group.

The Specific inclusion criteria are as follows: (a) Non-pregnant women aged between 18 and 40; (b) PCOS in strict accordance with 2.1.1 diagnostic criteria.

The specific exclusion criteria are as follows: (a) Age > 40 years old; (b) Pregnant women; (c) Suffering from other reproductive endocrine and metabolic diseases. On the basis of the above inclusion and exclusion criteria, the patients were divided into IR group and HA group according to HOMA-IR and HA values, and those who did not meet the diagnosis of IR and HA were in ELSE group.

(3) Control group: women of normal childbearing age who came to the hospital at the same time and passed the health examination were selected as the control group.

The specific inclusion criteria for the control group are as follows: (a) Non-pregnant women aged between 18 and 40 with regular menstruation and normal childbirth; (b) Patients without serious diseases of cardiovascular and cerebrovascular systems, digestive systems, liver, kidney and hematopoietic systems or mental disorders.

### Methods

#### Whole exome sequencing (WES)

Peripheral blood of PCOS family patients was collected for exon sequencing analysis, and was tested by the Novogene company.

1. Total DNA extraction.

- 200µL of EDTA whole blood was added to a 1.5mL EP tube, followed by the addition of protease K. The mixture was then thoroughly mixed and centrifuged.
- Buffer GB (200µL) was added to the mixture, which was subsequently mixed and centrifuged. The solution was then heated in a water bath at 70 °C until it became clear.
- Ethanol (200µL) was added to the solution, which was again mixed and centrifuged.
- The solution obtained from step c was transferred to an adsorption column and centrifuged at 12,000 rpm for 30 s. The waste liquid was discarded.
- Elution and centrifugation in the order of adding PW1, PW2, and PW1.
- The remaining liquid after step e was subjected to another round of centrifugation at 12,000 RPM for two minutes before discarding the waste liquid and allowing it to dry at room temperature.

i. DNA eluent TE (100µL) is added to obtain DNA solution, left at room temperature for five minutes, and then subjected to centrifugation at 12,000 rpm for two minutes.

j. Agarose gel electrophoresis is employed for analyzing DNA degradation level as well as detecting RNA and protein contamination presence.
k. Ultramicro nucleic acid detector was used for determining extracted DNA concentration after zeroing with TE buffer used previously; samples with concentrations exceeding 20ng/µl meet subsequent use requirements.

2. Build DNA library.

A DNA library was prepared by randomly interrupting the 180-280 bp segment of genomic DNA by Covaris fragmenter. After terminal repair and A-tail addition, a splice was attached to both ends of the fragment. After pooling, the library with specific index is hybridized with the biotin-labeled probe in liquid phase, and then streptomycin magnetic beads are used to capture the exon on the gene. After the library was constructed, Qubit 2.0 was used for preliminary quantification, and then NGS3K/Caliper was used to detect the insert size of the library. After the insert size met the expectation, the effective concentration (3 nM) of the library was accurately quantified by qPCR to ensure the quality of the library as well as subsequent sequencing. The Illumina platform was used for sequencing.

3. Data analysis and variation annotation.

After filtering the original data returned by sequencing, valid data were obtained, and sent to the company for reference genome, local reads comparison, de-duplication, base mass value re-correction, etc., to obtain the BAM analysis file and use multiple alignment processing to ensure the quality of SNP detection. The SNPS were detected and annotated by variation detection and software.

4. Screening of loci of variation.

The existing database, software and other tools were used to screen the mutation results step by step based on mutation frequency, functional region, harmful classification, recessive heredity pattern and so on, combined with sample information. Candidate susceptible mutation genes and loci were analyzed by Online Mendelian Inheritance in Man [29] (OMIM)database, which includes the information of known genetic diseases and corresponding susceptible genes, and American College of Medical Genetics and Genomics [30] (ACMG) mutation classification standard.

5. Functional annotation and analysis of candidate genes.

Gene ontology (GO), Kyoto Encyclopaedia of Genes and Genomes [31, 32] (KEGG), and disease ontology (DO) analyses were performed using the R, to analyse cell components of candidate genes function, molecular function, biological processes, pathogenic annotate, remove significantly related to family disease not mutation loci. Finally, the PCOS susceptibility genes and loci of this family were determined through literature review in the database as well as Sanger sequencing.

#### Determination of methylation degree of TGF-β1 gene: Bisulfite sequencing PCR (BSP)

1. Genomic DNA was extracted from the peripheral blood of each sample using a blood genomic DNA extraction kit (centrifugal column). The specific steps are the same as in 2.2.1 [1].
2. DNA bisulfite transformation and purification.
3. PCR.

a. Use http://www.ncbi.nlm.nih.gov/ to find the location and promoter sequence of human TGF-β1 gene on chromosomes online, and then use http://www.urogene.org/cgibin/methprimer/methprimer.cgi to design primers for BSP method of this gene online:

TGFB1- Forward: ATGGGGATATTATTTATAGTGGGGT.

TGFB1- Reverse: ACTCTTAACCACTATACCATCCTCC.

The amplified fragment was 201 bp in length and contained a total of 12 CpG loci.

Primitive sequence:

ATGGGGACACCATCTACAGTGGGGCCGACCGCTATCGCCTGCACACAGCTGCTGGTGGCACCGTGCACCTGGAGATCGGCCTGCTGCTCCGCAACTTCGACCGCTACGGCGTGGAGTGCTGAGGGACTCTGCCTCCAACGTCACCACCATCCACACCCCGGACACCCAGTGATGGGGGAGGATGGCACAGTGGTCAAGAGC.

If methylation occurs at the CpG site, after modification by bisulfite, it transforms into the following sequence:

ATGGGGATATTATTTATAGTGGGGTCGATCGTTATCGTTTGTATATAGTTGTTGGTGGTATCGTGTATTTGGAGATCGGTTTGTTGTTTCGTAATTTCGATCGTTACGGCGTGGAGTGTTGAGGGATTTTGTTTTTAACGTTATTATTATTTATATTTCGGATATTTAGTGATGGGGGAGGATGGTATAGTGGTTAAGAGT.

b. The extracted DNA was detected by ultra-micro accounting detector, and the required volume of DNA solution was calculated with the absolute value of 200ng. PCR reaction system was configured, and 3 groups were parallel (60µL for each sample).

c. The prepared reaction system was briefly centrifuged and then put into the PCR nucleic acid amplification apparatus.

(4) Transformation of recombinant DNA.

The DNA was transferred into competent cells through chemical transformation, enabling the growth of E. coli with the target plasmid on corresponding resistant plates. White colonies were selected for sample identification and subsequent sequencing. Five pairs of clones were selected from each sample.

(5) Result analysis.

BiQ Analyzer software was used to analyze the methylation of the sequencing results, and the methylation of each CpG site was obtained, as well as the histogram and dot graph.

#### RNA examination

1. Reverse transcription: Using purified peripheral blood RNA as template, cDNA was synthesized using Evo M-MLV reverse transcription reagent premix configuration system.
2. Three groups of replicates were set for each sample, and three samples with large standard deviation of Ct values were re-tested. 2^−△△CT^ formula was used to calculate the relative change of each gene expression in each sample.

#### Enzyme-linked immunosorbent assay(ELISA)

ELISA kit was used to detect the concentration of TGF-β1 by double-antibody sandwich method. Plasma TGF-β1 was captured by solid phase antibody labeled with horseradish peroxidase (HRP) to form antibody-antigen-enzyme-labeled antibody complex. After washing, substrate TMB (3,3’, 5,5’ -tetramethyl benzidine) was added to stain. TMB is converted to blue under the catalysis of HRP enzyme, and finally to yellow under the action of acid. The depth of the color was positively correlated with the concentration of TGF-β1 in the serum. The concentration was calculated using a standard curve using an Infinite M Plex spectrophotometer (Tecan, Mannedorf, Switzerland) with a wavelength of 450 nm.

#### Statistical analysis and image visualization

The data of this study was collated by Excel 2010, and statistical analysis was conducted by SPSS 22.0 software. Besides bioinformatics analysis, data image visualization was realized by GraphPad Prism 8, BiQ Analyzer. Bioinformatics analysis and visualization through R package ‘org.hs.eg.db’, ‘clusterProfiler’ [33] and ‘ggplot2’ [34] under R version 4.0.5.

Methylation Level (%) = Number of methylated samples at this site/total number of samples * 100%.

## Results

### Basic family information

Three women in the family were diagnosed with PCOS, I-2, II-2, and II-5, while the phenotype of III-3 was unknown. As shown in Fig. 1, the mother had PCOS, and her three daughters were all diagnosed with PCOS. Two were identical twins; therefore, only one was included in the WES. It showed that the body mass index (BMI) of each family member was within the normal range, whereas the insulin resistance index (HOMA-IR) of the three PCOS patients was higher than normal, indicating that the patients in the family had different degrees of insulin resistance (Table 1).

**Table 1:** Basic clinical characteristics of PCOS family members

Family member number	I-2	III-3	II-2	II-5
Gender	Female	Female	Female	Female
Age	69	12	25	22
BMI	19.3	22.4	20.6	23.7
FSH (mIU/ml)	42.1	3.21	5.76	6.08
E2 (pmmol/L)	10.5	24	92.7	79.50
T (nmol/L)	0.54	1.24	1.34	1.04
HOMA_IR	2.81	2.01	3.26	2.7
Diagnosis	PCOS	Unknown	PCOS	PCOS

### WES screening results

#### Quality of sequencing data

The data quality was analysed using data from the Illumina double-ended sequencing, as shown in Table 2. Q20 was greater than 97%, Q30 was greater than 93%, and the average error rate was less than 0.03%; this is far better than the minimum standard for WES sequencing results, indicating reliability and potential utility in subsequent analyses.

**Table 2:** Output quality of WES data

Sample	Raw reads ^a^	Raw data ^b^ (G)	Raw depth ^c^ (x)	Effective ^d^ (%)	Error ^e^ (%)	Q20 ^f^ (%)	Q30 ^g^ (%)
I-2	34,728,761	10.42	172.35	96.61	0.03	97.89	93.95
III-3	43,235,056	12.97	214.53	95.72	0.025	97.97	94.16
II-2	35,067,419	10.52	174.01	98.78	0.03	97.82	93.91
II-5	33,787,670	10.14	167.72	98.19	0.03	97.78	93.83

^a^ Total number of read pairs at both ends of the original sequence (log of reads). ^b^ The amount of data per sample. ^c^ The amount of data per sample. ^d^ Ratio of filtered reads to data volume. ^e^ Average error rate for all bases. ^f^ The percentage of bases with sequencing base quality values greater than 20 from the total bases was calculated. ^g^ The percentage of bases with sequencing base quality values greater than 30 from the total bases was calculated

#### Statistics of sequencing depth and fraction of target covered

The reference genome (GRCh37/ hg19) was compared with the effective reads of the four samples (I-2, II-3, II-2, and III-5) after repeated removal, and the fraction of the target covered by the sample sequencing data was determined, as shown in Table 3. The mapping rates were 99.92%, 99.93%, 99.93%, and 99.94%, respectively. The average coverage depths of exons in the target region were 107.66x, 135.16x, 123.71x, and 117.01x, and the fraction of targets covered with at least 10x reached 97.6%, 98.2%, 97.4%, and 97.0%, respectively. Based on the comprehensive judgment of the summary results of sample sequencing quality, the statistical results of sequencing depth, and the fraction of target covered, the sample data met the analysis requirements and could be further analysed.

**Table 3:** Statistics of sequencing depth and fraction of target covered

Sample	I-2	III-3	II-2	II-5
Total reads	67,101,716	82,770,842	69,279,058	66,350,348
Duplicate reads (Rate: duplicate reads/clean reads)	14,421,085 (21.51%)	19,975,421 (24.15%)	12,631,824 (18.25%)	10,389,088 (15.67%)
Mapped reads ^a^	67,051,047 (99.92%)	82,712,631 (99.93%)	69,231,702 (99.93%)	66,308,692 (99.94%)
Average sequencing depth on target ^b^	107.66	135.16	123.71	117.01
Fraction of target covered with at least 10x ^c^	97.6%	98.2%	97.4%	97.0%

^a^ Total number of reads aligned to reference genome (proportion). ^b^ Average sequencing depth of target region (total data volume aligned to target region/total length of target region). ^c^ Proportion of bases in the target region that cover a depth of not less than 10X

#### SNP variation results

Based on these reliable results, sequence Alignment/mapping **(**SAM) tools were used to identify and filter SNP sites. The ANNOVAR software annotated the SNP variation location, type, conservatism, and other indicators (Table 4).

**Table 4:** Number of different types of SNP in this family

Sample	synonymous SNP^a^	missense SNP^b^	stopgain^c^	stoploss^d^	unknown
II-5	11,107	10,021	77	10	450
II-2	11,089	9,995	73	11	424
I-2	11,041	10,041	70	10	460
III-3	11,026	10,001	68	8	436

^a^ The amino acids encoded by the mutation sites did not change. ^b^ The amino acids encoded by the mutation site changed, which was a non-synonymous mutation. ^c^ Substitution of a base causes the codon in which the base resides to become a stop codon. ^d^ Substitution of a base causes the termination codon in which the base resides to become a non-termination codon

#### Screening results

SNP information obtained from the analysis was screened (Supplementary Fig. 1).

#### ACMG hazard classification of mutated sites

The gold standard for interpreting data after high-throughput sequencing is based on the ACMG standards and guidelines for predicting how harmful mutations are. The guide divides the variation into pathogenic, likely pathogenic, uncertain significance, likely benign, and benign according to the combination of 28 categories of evidence; the five categories are used to describe mutations found in Mendelian disease genes [30] (Table 5). There were three predicted pathogenic loci, 23 possibly pathogenic loci, 2090 loci of unknown pathogenicity, 1587 possibly benign loci, and 28,282 benign loci; altogether, there were 28,282 mutation loci. Our subsequent research and analysis focused on the genes corresponding to the three loci of non-benign variation.

**Table 5:** ACMG hazard classification of mutated sites

Total	pathogenic	likely pathogenic	uncertain significance	likely benign	benign
31,985	3	23	2090	1587	28,282

#### Genetic pattern screening

In this study, three members of this family were classified, discussed, and analysed, and the results were collected. If the susceptibility gene for PCOS followed a dominant inheritance pattern, we screened the SNP loci in the lines of the corresponding 71 candidate genes. If the PCOS susceptibility gene had a recessive inheritance pattern, including homozygous and compound heterozygous mutations, in this study, no eligible genes were screened.

#### Bioinformatic candidate gene enrichment analysis

**Fig. 2:**
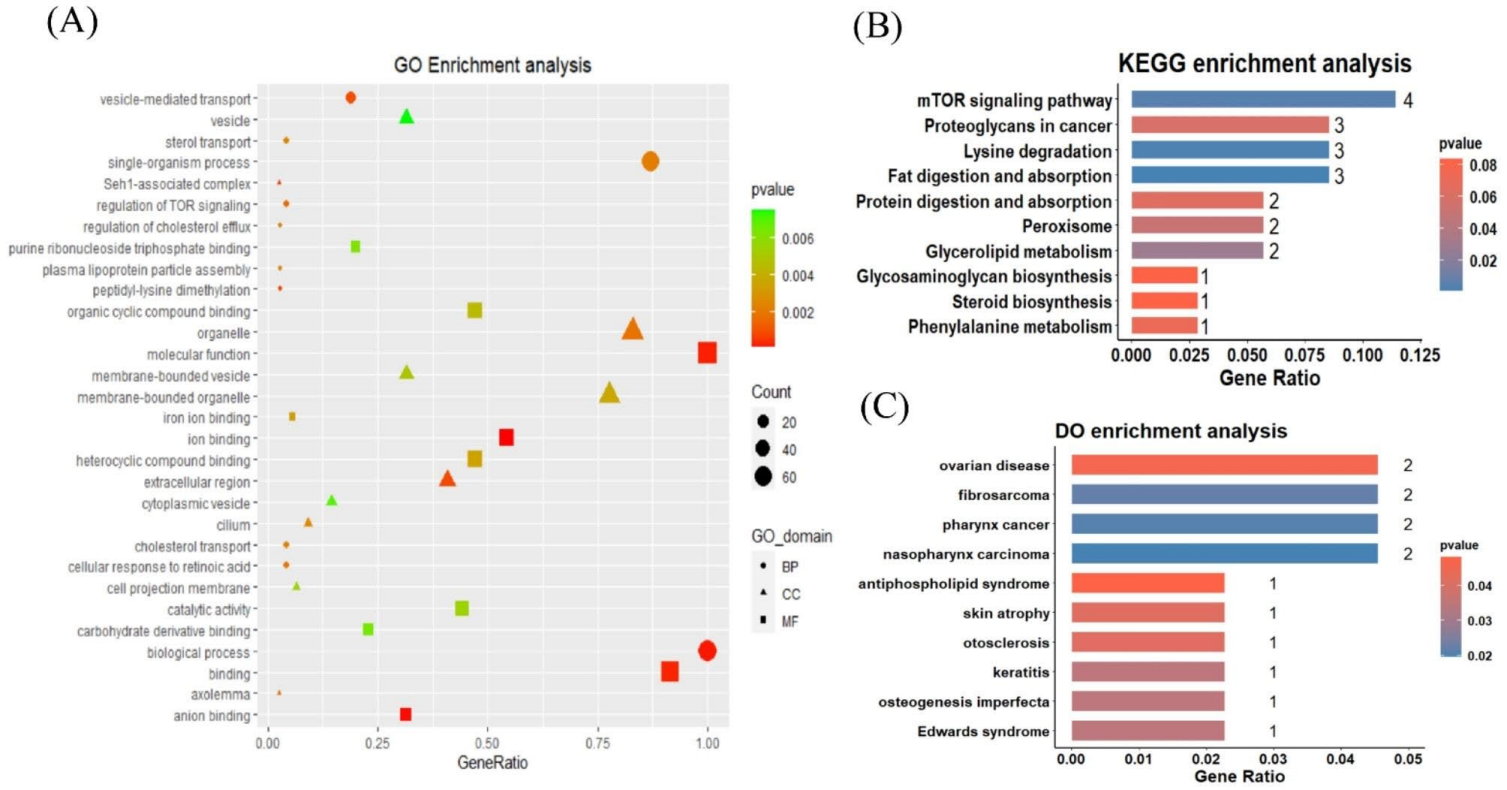
Candidate gene enrichment analysis figure. (**A**) Part of resulting diagram of GO enrichment analysis of candidate genes. The pattern shape indicates annotation of gene ontology, the pattern size indicates number of enriched genes, and pattern colour indicates size of the p-value. (**B**) Part of the KEGG enrichment analysis of candidate genes. Abscissa indicates number of enriched genes, and colour indicates size of p-value. (**C**) Partial results of DO analysis of candidate genes. Abscissa indicates number of enriched genes, and colour indicates size of p-value

Candidate genes were functionally enriched in the GO, KEGG, and DO analyses. The GO analysis candidate genes were mainly related to biological functions such as vesicle transport, sterol transport, TOR signalling regulation, cholesterol efflux regulation, purine riboside triphosphate binding, and plasma lipoprotein particle assembly (Fig. 2A and Supplementary Table S1). The KEGG analysis candidate genes were mainly related to the mTOR, fat digestion and absorption, protein digestion and absorption, peroxisome signalling pathways, and glycerol metabolism (Fig. 2B and Supplementary Table S2). The DO analysis candidate genes were mainly associated with reproductive and immune diseases, such as ovarian disease, fibrosarcoma, antiphospholipid syndrome, skin atrophy, otosclerosis, and osteogenesis (Fig. 2C and Supplementary Table S3).

#### PCOS susceptibility gene pedigree

Based on the above results, Sanger sequencing and the PubMed, NCBI, and other databases, we selected some genes highly likely to be associated with PCOS in this family (Table 6): *DGAT1, EHMT1, KDR, LAMTOR1, SEC13, SETD2*, and *TGF-β1.* In order to further explore the association between susceptibility genes and PCOS, we set the inclusion criteria for the following genes: (a) In The Genome Aggregation Database, the allele frequency of the mutated base (GnomAD_ALL_AF) was greater than 0.0002. (b) Five or more conservative annotation software suggest possible harm (Supplementary Table S5). Through the inclusion and exclusion criteria, we finally selected *TGF-β1* as the object of our study.

**Table 6:** List of SNP candidate susceptibility genes screened out

Variation Type		Gene Name	Position^a^	ID	REF^b^	ALT^c^	GnomAD ALL AF^d^	AA Change^e^	cytoband^f^
SNV	DGAT1		chr8:144318530	rs144065666	C	T	0.00017943	NM_012079:exon6:c.G505A:p.V169M	8q24.3
SNV	EHMT1		chr9:137777959	rs141689686	C	T	0.00001804	NM_001145527:exon13:c.C2096T:p.T699M	9q34.3
SNV	KDR		chr4:55115364	rs35636987	C	T	0.00068263	NM_002253:exon4:c.G406A:p.V136M	4q12
SNV	LAMTOR1		chr11:72098308	rs146341570	G	A	0.00043963	NM_017907:exon4:c.C374T:p.P125L	11q13.4
SNV	SEC13		chr3:10305050	rs191151688	T	C	0.00001625	NM_001136232:exon7:c.A649G:p.I217V	3p25.3
SNV	SETD2		chr3:47124067	rs563907746	G	A	0.00011469	NM_001349370:exon2:c.C437T:p.P146L	3p21.31
SNV	TGF-β1		chr5:136055770	rs121909212	C	A	0.00029683	NM_000358:exon11:c.C1501A:p.P501T	5q31.1

^a^ The absolute chromosomal position of a locus of variation. ^b^ Reference genome base type. ^c^ Sample genome base type. ^d^ Allele frequencies of the mutated base at this variant locus in all populations. ^e^. Amino acid change. ^f^. The chromosomal segment on which the mutation occurs

### Basic PCOS sample information

To study the effects of DNA methylation of the *TGF-β1* gene on PCOS, clinical volunteers were selected according to strict inclusion and exclusion criteria to exclude patients with multiple phenotypes involving pregnancy, abortion, drugs, and other possible influencing factors. Twenty-eight healthy women, 13 patients with IR PCOS, 12 patients with HA PCOS, and six patients with neither IR nor the additional phenotype of HA were selected. The clinical indicators used in this study are listed in Table 7.

**Table 7:** Basic clinical characteristics of selected clinical samples

Group	CONTROL (n = 28)	IR (n = 13)	HA (n = 12)	ELSE (n = 6)	p value
Age	30 ± 4	27 ± 3	28 ± 3	30 ± 4	0.104
Height (m)	1.57 ± 0.05	1.57 ± 0.05	1.54(0.02)	1.56 ± 0.04	0.285
Weight (kg)	50.9 ± 7.2 a	59.3 ± 11.1 b	53.1 ± 2.8 a, b	48.9 ± 4.5 a	0.008
BMI	20.68 ± 2.40 a	23.92 ± 3.74 b	22.30 ± 1.27 a, b	20.03 ± 2.38 a	0.002
Testosterone (T) (nmol/L)	1.06 ± 0.42 a	1.15 ± 0.32 a	2.1 (0.69) b	1.35 (0.30) a, b	< 0.001
Fasting plasma glucose (nmol/L)	5.05 ± 0.66	5.02 ± 0.68	4.89 ± 0.63	5.32 ± 0.45	0.621
Fasting insulin (uU/ml)	9.56 ± 2.9a	19.5 ± 8.77 b	10.8 ± 2.22 a	10.18 ± 1.01a	< 0.001
Homa-IR	2.27 (0.86) a	4.51 ± 2.37 b	2.33(0.56)a, b	2.40 ± 0.24 a, b	0.002

a, b: If two groups had the same marker letters, there was no statistically significant difference between them. If the two groups had different letters, the difference between them was considered statistically significant. (Hereafter, this is the same.)

### DNA methylation of *TGF-β1* by bisulfite sequencing

**Fig. 3:**
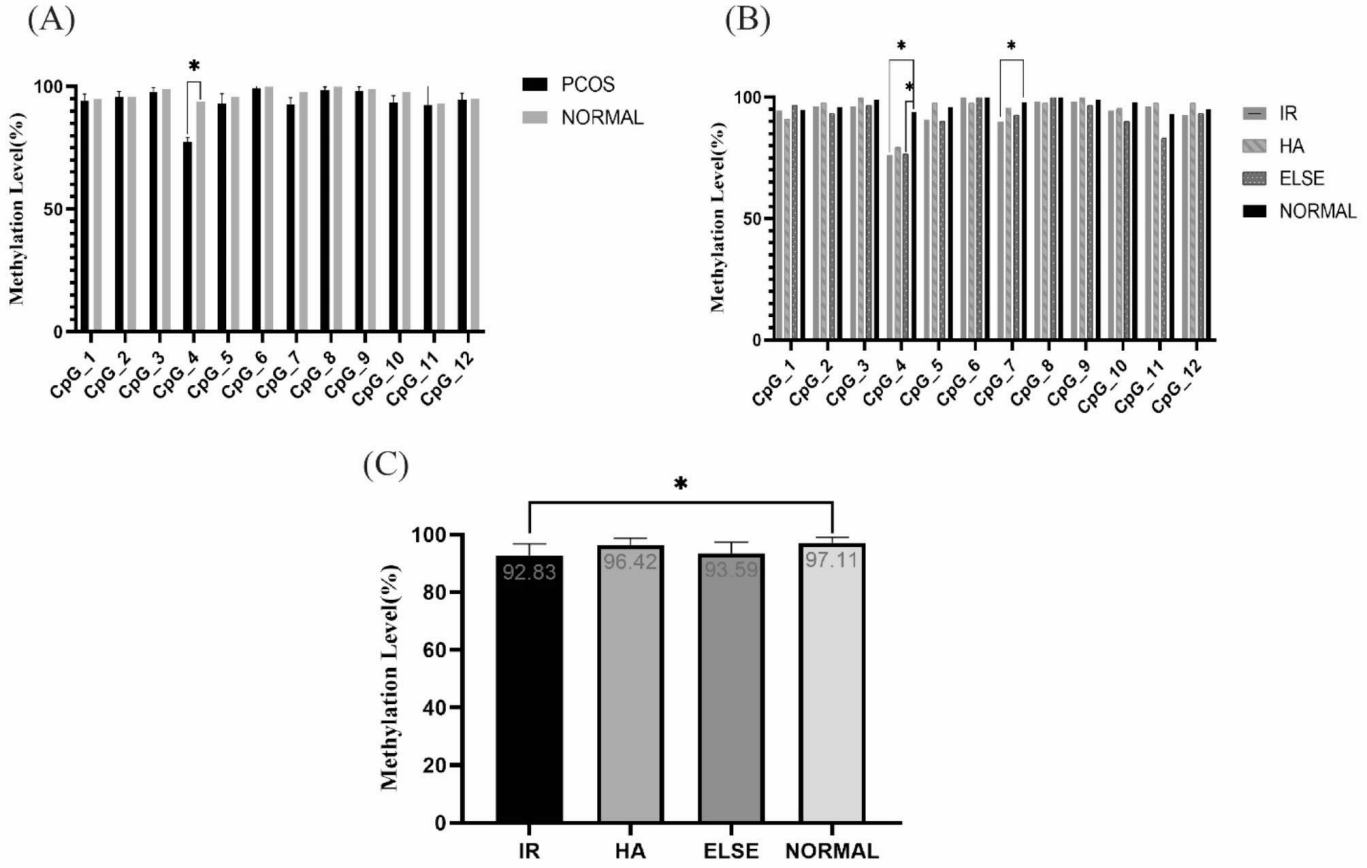
(**A**) *TGF-β1* CpG loci gene promoter methylation rate of PCOS patients and normal control group graph. (**B**) Bar diagram of methylation rates of CpG sites of *TGF-β1* gene promoters under different phenotypes. (**C**) Bar diagram of total methylation rates of all CpG sites of *TGF-β1* gene promoters

After TA cloning and sequencing, and BiQ Analyzer analysis, bar charts and dot charts were generated after independent analysis of a single sample (Supplementary Fig. 2). Panel A is the bar chart of methylation sites, blue represents unmethylated sites, and yellow represents methylated sites. Panel B is a dot plot with solid circles indicating unmethylation and hollow circles indicating methylation occurring. A comprehensive analysis of all samples revealed that among the 12 CpG sites, CpG4 was significantly hypomethylated in PCOS patients compared with those in normal controls (p = 0.001) (Fig. 3A). The methylation levels of two loci differed among the four groups: CpG4 (p = 0.004) and CpG7 (p = 0.046). Pairings showed significant hypomethylation of CpG4 (p = 0.004) and CpG7 (p = 0.012) in the IR group compared with the normal control group, with a calibrated test level α = 0.0125. In addition, the methylation level of CpG4 in the ELSE group of PCOS patients was significantly lower than that in the control group (p = 0.012) (Fig. 3B). There were differences in total methylation rate between groups (p = 0.004), and the paired comparison showed that the *TGF-β1* gene promoter methylation level in the IR group was significantly lower than that in the normal group (p = 0.005) (Fig. 3C).

### Correlation between DNA methylation of *TGF-β1* and clinical data

**Fig. 4:**
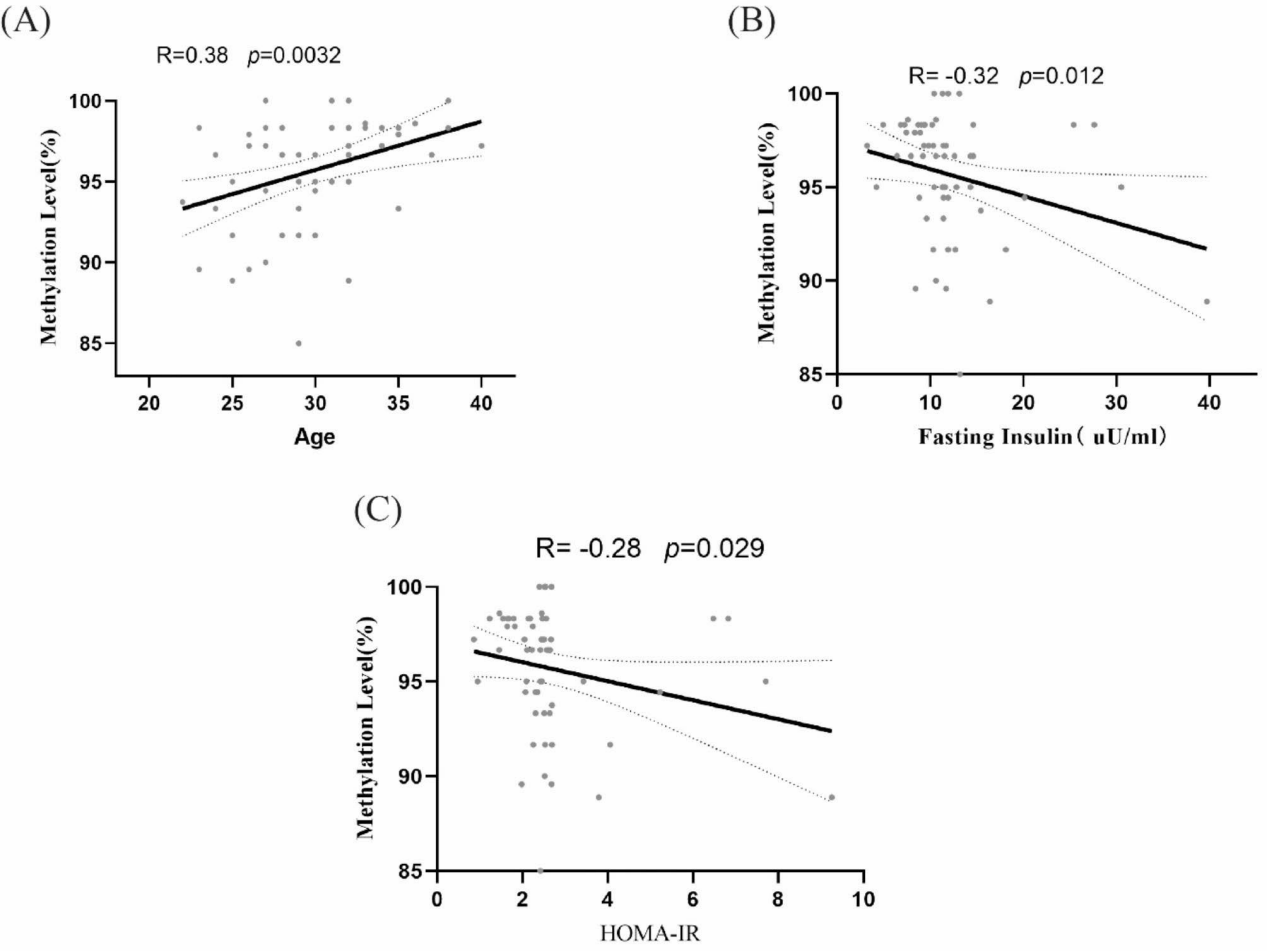
(**A**) Correlation between age and methylation rate. (**B**) Correlation between fasting insulin and methylation rate. (**C**) Correlation between HOMA-IR and methylation rate

Each sample was regrouped according to methylation levels (Supplementary Table S4). The correlation between the methylation rate and clinical indicators was further explored. It was found that methylation rate was positively correlated with age (R = 0.38, p = 0.0032) and negatively correlated with fasting insulin and HOMA-IR (R = -0.32, p = 0.012; R = -0.28, p = 0.029), (Table 8; Fig. 4).

**Table 8:** Correlation between methylation rate and clinical indicators

	Age	BMI	T	Fasting plasma glucose	Fasting insulin	HOMA-IR
R	0.38	-0.1003	0.05	0.03	-0.32	-0.28
p-value	0.0032	0.45	0.69	0.82	0.012	0.029

**Fig. 5:**
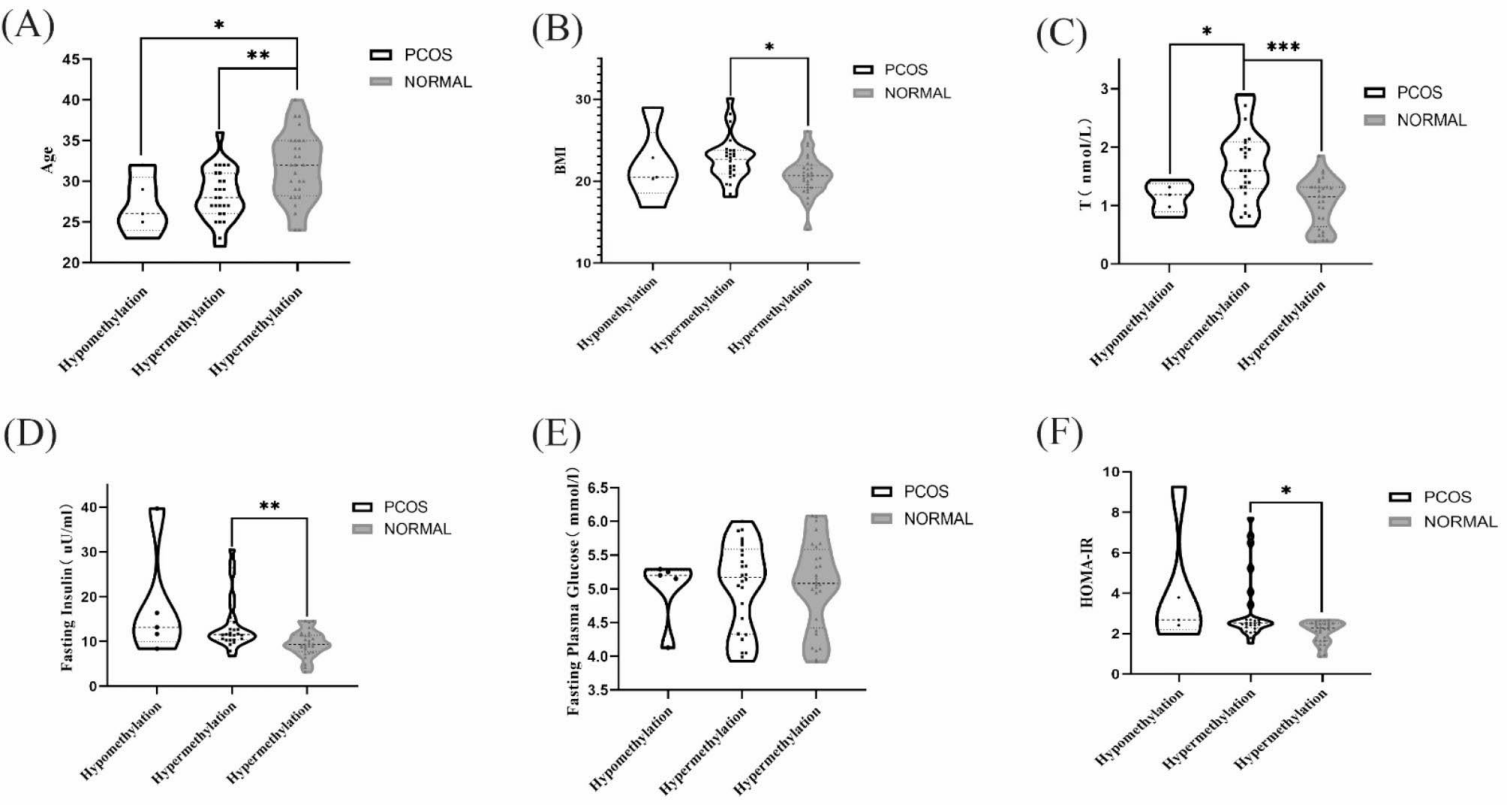
(**A**) Violin diagram of age differences between different subgroups. (**B**) Violin diagram of BMI differences between different subgroups. (**C**) Violin diagram of testosterone differences between different subgroups. (**D**) Violin diagram of fasting plasma glucose differences between different subgroups. (**E**) Violin diagram of fasting insulin differences between different subgroups. (**F**) Violin diagram of HOMA-IR differences between different subgroups

The PCOS and normal control groups were stratified according to total methylation levels at all sites. The methylation rates in the control group were all > 90%. Therefore, according to the cut-off value of 90%, the PCOS group was divided into hypomethylated (ML < 90%) and hypermethylated (ML > 90%) groups. All the healthy controls were included in the same group (Table 9). The difference in the clinical data of each group was studied, and it was found that in PCOS patients, the testosterone level in the high methylation rate group was significantly higher than that in the low methylation rate group. Under the high methylation rate, the BMI, testosterone level, fasting insulin level, and HOMA-IR of the normal control group were significantly lower than those of the PCOS patients, but their age was significantly higher than that of the PCOS patients (Fig. 5).

**Table 9:** Samples of each group stratified and grouped according to the degree of methylation

Group	PCOS		NORMAL	p-value
	Hypomethylation	Hypermethylation		
Methylation rates	85.00 − 90%	90.00 − 100.00%	90.00-100.00%	-
Number (N)	5	26	28	-
Age	27.0 ± 3.5 a	28.3 ± 3.2a	31.7 ± 4.2b	0.002
BMI	21.88 ± 4.51 a, b	22.66 (2.88) a	20.68 ± 2.40b	0.028
T (nmol/L)	1.15 ± 0.26 a	1.69 ± 0.63b	1.06 ± 0.42a	< 0.001
Fasting plasma glucose (mmol/l)	5.20(0.63)	5.04 ± 0.65	5.05 ± 0.66	0.992
Fasting insulin (uU/ml)	13.20 (18.00) a, b	11.55 (4.20) a	9.56 ± 2.90b	0.002
Homa-IR	4.03 ± 3.00 a, b	2.53 (0.58) a	2.28 (0.85) b	0.008

### TGF-β1 RNA and protein expression

**Fig. 6:**
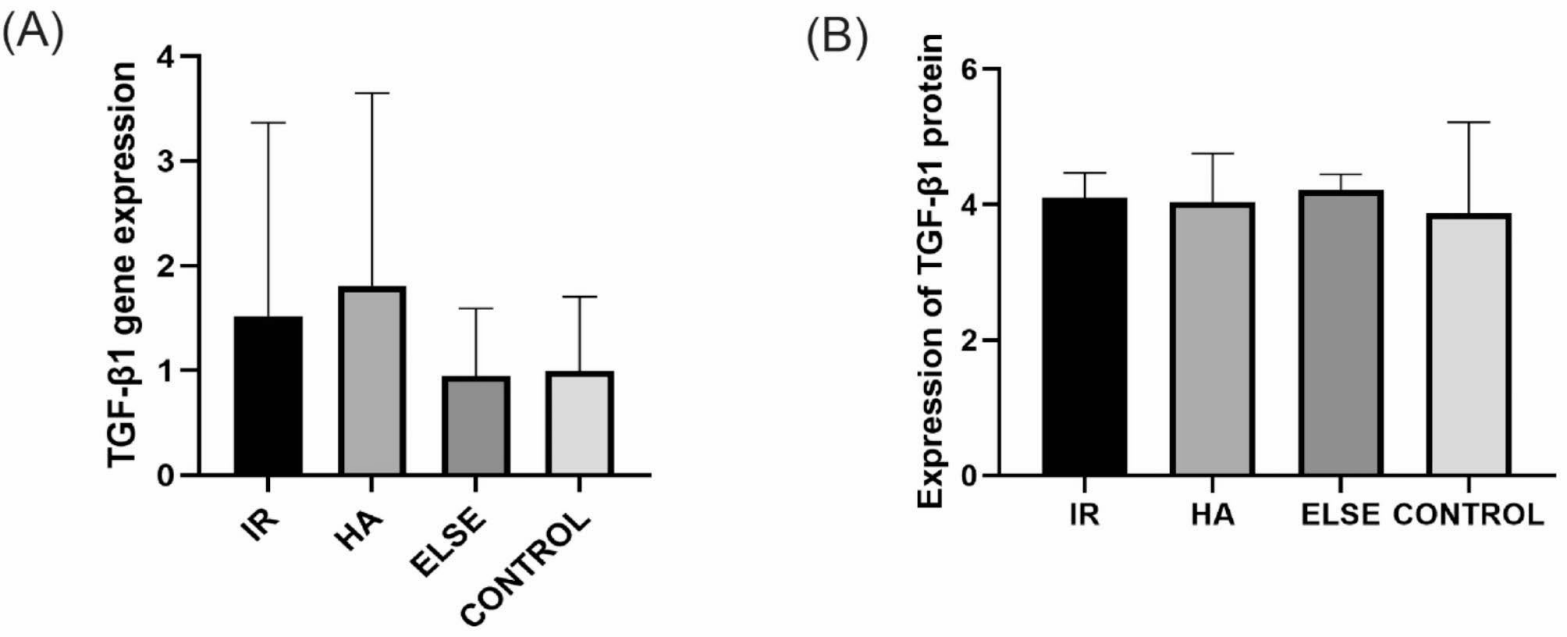
(**A**) Histogram of mRNA relative expression of *TGF-β1* gene. (**B**) Histogram of TGF-β1 protein expression

RT-PCR was used to study the mRNA expression of *TGF-β1* in clinical volunteers. The results showed that compared with the normal control group, the expression level of *TGF-β1* in the IR and HA groups was higher, but the results showed no statistical difference. The relative expression level was 0.66 (2.13) in the IR group and 1.809 ± 1.838 in the HA group, p = 0.817 (Fig. 6A). Further, it was found that there was no significant correlation between methylation rate and mRNA expression level (R = 0.24, p = 0.24).

The expression level of TGF-β1 in the serum samples of clinical volunteers was detected by the ELISA method (Fig. 6B), and no significant difference was found between the four groups. For the IR group, it was 4.11 ± 0.36; for the HA group, it was 4.03 ± 0.72; for the ELSE group, it was 4.22 ± 0.23; for the NORMAL group, it was 4.32 (1.31), p = 0.96. Further exploring the correlation between methylation rate and TGF-β1 protein expression, it was found that there was no significant correlation between methylation rate and TGF-β1 protein expression (R = -0.29, p = 0.08).

## Discussion

PCOS is an endocrine disease with a high incidence in women of childbearing age. Its basic pathophysiological features include HA and insulin resistance caused by disturbances in the ovarian environment, cytokine expression, and dysfunction. At present, the aetiology of PCOS is still not completely clear; however, based on existing studies, it is caused by environmental and genetic factors. As a disease with complex aetiology, PCOS may be caused by the joint action of multiple genes and their mutations or polymorphisms [6].

### Single nucleotide variation in susceptibility genes in the PCOS family

In this study, the susceptibility genes of the family of PCOS patients were screened using whole-exon sequencing technology for the first time. Seven susceptibility genes were predicted: *TGF-β1, DGAT1, EHMT1, KDR, LAMTOR1, SETD2*, and *SEC13*.

In the past, genome-wide association studies (GWAS) have often been used to study the genetics of complex diseases. However, this method has several significant shortcomings. First, due to the inclusion of the whole genome, the identified gene loci are often located in non-coding or non-functional regions, making it difficult to explain the specific mechanisms underlying the occurrence of diseases. Second, the time taken limits the use of GWAS. The expressed region is the part of the eukaryotic DNA expressed as a protein. Exons account for approximately 1% of the human genome; however, 85% of disease-causing mutations occur in this region [35]. Therefore, whole-exome sequencing, through the capture, enrichment, and high-throughput sequencing of exons, provides a new, economical, and efficient method for identifying susceptibility genes.

Traditional case-control studies are prone to bias due to factors such as race, environment, and genetic background affecting the results. As PCOS is a genetically predisposed disease, Studies on the correlation between susceptibility gene loci and disease based on family pedigree can ensure consistency in terms of genetic background, living habits, and environment, eliminating the interference of confounding factors. Therefore, we selected a small sample from this family to study the susceptibility genes of PCOS, which can guide subsequent research to a certain extent.

In view of the wide range of biological functions of *TGF-β1* and the diverse clinical manifestations of PCOS, research has explored their relationship. Many studies have shown that abnormal *TGF-β1* expression is involved in multiple pathological PCOS changes and has foetal origins [36, 37]. *TGF-β1* is associated with the pathological manifestations of ovarian fibrosis, HA, ovulation disorders, and insulin resistance equal to that in PCOS [9, 14–19, 22, 23, 38, 39]. However, most of these studies have focused only on the level of correlation without studying its mechanism from the perspective of the pathway.

TGF-β intracellular signal transduction relies on the Smads protein family, which directly or indirectly acts on target genes and affects the transcription and expression of downstream genes [40]. This series of regulatory processes has gradually become a focus of research. the TGF-β1 protein and a variety of cytokines communicate in the TGF-β/Smads pathway, which is involved in the growth and development of the body and a variety of diseases in the process of bidirectional regulation [41]. Integrating the references, we proposed hypotheses explaining how mutations in the TGF-β1 gene could lead to insulin resistance (Supplementary Fig. 3).

Recently, it has been found that kisspeptin (KP), a hypothalamic peptide encoded by the *KISS 1* gene, may act as a key mediator of the hypothalamic-pituitary-gonadal axis, any small deviation of KP signal may lead to the occurrence of complex reproductive diseases [42].Tian et al. [43]. reported that *KISS 1* is the downstream target of the classical TGF-β1/Smad2 signaling pathway. Fang et al. [44]. demonstrated that TGF-β1 upregulates the expression of KP in human extravillous cytotrophoblast cells. The induction of KP expression by TGF-β1 occurs through a Smad-independent ERK 1/2 signaling pathway.

KP may promote the occurrence of PCOS by exerting an influence on gonadotropin-releasing hormone neurons, thereby stimulating luteinizing hormone secretion and causing ovulation disorders [45]. Additionally, KP level have been found to be associated with glucolipid metabolism; however, the causal relationship remains unclear. The similarities in pathways and characteristics between KP and TGF-β1 suggest that the interaction between these two factors may play a crucial role in the development and progression of PCOS. Further research is warranted to explore novel therapeutic targets for reproductive disorders such as PCOS and endometriosis.

In the screening of susceptibility genes in families by exon sequencing, it was found that *DGAT1, EHMT1, KDR, LAMTOR1, SETD2*, and *SEC13* may be susceptibility genes for PCOS. *DGAT1* encodes diacylglycerol o-acyltransferase 1, a multichannel transmembrane protein and key metabolic enzyme possibly associated with obesity and other metabolic diseases. EHMTI is a histone methyltransferase regulating the monomethylation and dimethylation of histone H3 lysine 9 (H3K9), which forms heteropolymeric complexes with G9a in euchromatin. EHMT1 has various functions and is associated with tumour development, obesity, embryo growth, and cardiac hypertrophy. The brown adipose-specific loss of EHMT1 results in a significant reduction in tissue-mediated adaptive thermogenesis, obesity, and systemic IR [46]. *KDR* encodes a type 2 receptor for vascular endothelial growth factor (VEGF). VEGF is highly specific and can promote the growth of vascular endothelial cells, increase vascular permeability, and promote the degeneration of the extracellular matrix. The VEGF system has been linked to ovarian diseases, including PCOS, in which follicles are prevented from developing [47–51]. LAMTOR1, a late endosomal/lysosomal connector, which acts as a MAPK and MTOR activator 1, plays important roles in energy and glucose metabolism. Huang et al. [52] found that mouse models with β-cell *LAMTOR1* gene-specific defects have higher glucose tolerance and glucose-stimulated insulin secretion during hyperglycaemic clamp and islet perfusion than control models. LAMTOR1 loss increases the amplification pathway induced by glutamic acid and acetyl-CoA carboxylase 1, ultimately leading to increased insulin secretion. The histone lysine methyltransferase SETD2 regulates the trimethylation of lysine 36 (H3K36) in histone H3 and is involved in the maintenance of chromatin structure, transcriptional extension, and genome stability. The proteins encoded by *SEC13* belong to the WD(Trp-Asp) repeat protein family, a component of the endoplasmic reticulum and nuclear pore complex and are required for endoplasmic reticulum vesicle biogenesis during transport [53]. Section 13 has also been linked to inflammation in the body [54], Macrophages of *SEC13* mutant mice expressed low levels of MHC I and II and had high levels of soluble and membrane-bound TGF-β and serum immunoglobulin production. TGF-β expression remained high after stimulation or immunisation, suggesting that *SEC13* is a key influencing factor in TGF-β production.In the family included in this study, both *SEC13* and *TGF-β1* genes were mutated, and it is speculated that the genes interact through their independent changes, with noteworthy changes in the transport of substances in vivo and immune function.

At present, studies on PCOS and the above-mentioned genes are limited. Subsequent studies can serve as a bridge between insulin resistance and abnormal lipid metabolism and further elucidate the aetiology and pathogenesis of PCOS by focusing on the mechanism of action of related genes. Complex diseases usually have multiple etiologies. Although it is impossible to explain the pathogenesis by single or a few genes with simple heterozygous mutations, we still hope to identify the SNP sites most likely to affect the family through bioinformatics analysis and the guidance of existing research, so as to provide a good direction for further research on this topic.

### *TGF-β1* gene promoter methylation and PCOS

Regarding the aetiology and pathogenesis of PCOS, epigenetics can better explain the comprehensive effects of heredity, environment, nutrient metabolism, and neuroendocrine regulation, a new consensus reached by the academic community [55]. Epigenetic modifications are reversible and adjustable. Compared with the direct intervention of gene expression, it has greater development potential and is likely a means to study the pathogenesis and intervention of diseases; it has gradually been applied in diabetes, hypertension, obesity, and other metabolism-related diseases [7]. Based on existing studies, TGF-β1 is involved in the regulation of follicular growth, ovarian fibrosis, and insulin resistance, affecting the occurrence and development of PCOS. Therefore, *TGF-β1* was selected as the susceptibility gene to further investigate the influence of methylation of this gene on the occurrence and development of PCOS.

The DNA methylation epigenetic modification of *TGF-β1* has been studied in many fibrosis-related diseases such as lung, liver, and heart diseases [56–58]. Nour et al. [59] reported for the first time that neuroendocrine and metabolic abnormalities in PCOS mice could be significantly improved by applying the methylation-promoting agent S-adenosyl methionine to their progeny.

In the PCOS rat model, our team found that the DNA methylation of the anti-Mullerian hormone and insulin receptor genes in blood lymphocytes was closely related to ovarian pathological changes, ovulation disorders, and insulin resistance [60, 61], However, no studies on *TGF-β1* methylation and PCOS pathological manifestations have been reported. Tian et al. [62] used letrozole to induce PCOS in a rat model; the results showed that *TGF-β1* expression levels in the follicular membrane and interstitial cells were significantly higher than those in the control group. However, the specific mechanism affecting its expression was not clear, and there is no study on whether the same is true in peripheral blood.

In this study, the *TGF-β1* gene promoters in the 59 subjects showed significant CpG4 hypomethylation in PCOS patients compared with the normal controls (p = 0.001). The methylation rates of the CpG4 (p = 0.004) and CpG7 (p = 0.012) sites in the IR group were significantly lower than those in the normal control group. In addition, the CpG4 methylation level in the ELSE group of PCOS patients was significantly lower than that in the control group (p = 0.012). The total methylation rate in the IR group was significantly lower than that in the normal group (P = 0.005), and the methylation rate was positively correlated with age (R = 0.38, p = 0.0032) and negatively correlated with fasting insulin and HOMA-IR (R = -0.32, p = 0.012; R = -0.28, p = 0.029). There was no significant difference in mRNA and protein expression levels between the different groups of *TGF-β1* methylation level, and no correlation with methylation rate. Subgroup analysis of the methylation levels showed that the testosterone level in the high methylation rate PCOS group was significantly higher than that in the low methylation rate PCOS group. Under the high methylation rate, the BMI, testosterone level, fasting insulin level, and HOMA-IR of the normal control group were significantly lower than those of PCOS patients, but their age was significantly higher than that of patients with PCOS. There was no significant difference in mRNA and TGF-β1 protein expression between these groups.

In this study, we found that methylation at the CpG4 site of the *TGF-β1* promoter may affect PCOS pathogenesis and is more likely to cause insulin resistance than HA; the methylation rate is negatively correlated with fasting insulin level and HOMA-IR in the population. It is generally believed that hypermethylation inhibits transcription and hypomethylation promotes transcription [63], In the insulin resistance group, there are two low methylation CpG loci, the ELSE group have a CpG locus with low methylation, but did not make corresponding mRNA, and protein expression was significantly higher, on the one hand, may be associated with overall degree of methylation of the promoter, two loci methylation differences do not affect the transcription of the promoter area; this may be because the mRNA expression level is also affected by other factors, such as non-coding RNA and gene copy number variation. Additionally, BSP was used to measure methylation. Differences in each independent CpG locus were studied, easily leading to significant but small differences in the degree of methylation at the overall gene level, which failed to have a significant impact on the subsequent mRNA expression level. According to the results of this study, we speculate that abnormal methylation of *TGF-β1* in peripheral blood may not be the direct cause of PCOS. However, due to the tissue specificity of DNA methylation and the interaction of genes, the epigenetic role of the *TGF-β1* gene in PCOS cannot be completely denied. This study found that in women of childbearing age, the higher the methylation rate of the *TGF-*β1 gene, the lower the fasting insulin level and insulin resistance index, which also reflected the effect of the methylation rate on the IR phenotype.

Subgroup analysis of the case and control groups according to the methylation level showed that in patients with PCOS, the testosterone level in the high methylation rate group was significantly higher than that in the low methylation rate group. The reason for this result in our study is probably that the PCOS patients in the hypomethylation group did not have HA symptoms; all came from the IR and ELSE groups. This may indicate that the degree of methylation of the *TGF-β1* gene promoter may not be directly associated with HA.

We also noted that methylation rates positively correlated with age in women of childbearing age (R = 0.38, p = 0.0032). This suggests that the lower the age, the lower the methylation rate of the *TGF-β1* gene in peripheral blood during the reproductive age, which may increase the incidence of PCOS, especially IR PCOS. This is consistent with PCOS epidemiology in that the high incidence is concentrated in 18–30-year-olds [64]. This also suggests that early PCOS prevention and intervention is particularly important as the methylation degree of *TGF-β1* in peripheral blood may increase with age, and the role of *TGF-β1* in PCOS pathogenesis may gradually decrease. However, this requires further verification in population cohort studies.

Inevitably, there are some drawbacks in this study. Firstly, we only included women in one PCOS family for sequencing, which lacks a large population study on the susceptibility genes. Secondly, PCOS susceptibility sites may also be distributed in the intron region of genes, producing regulatory non-coding RNA that affect protein expression. This is also a disadvantage of WES. In addition, although we found an association between *TGF-β1* methylation and PCOS phenotypes, the mechanism has not been further explored and further studies are needed.

### Electronic supplementary material

Below is the link to the electronic supplementary material.

Supplementary Material 1

Supplementary Material 2

Supplementary Material 3

Supplementary Material 4

## Data Availability

The datasets generated and/or analysed during the current study are available in the NCBI BioProject epository, PRJNA986898 https://www.ncbi.nlm.nih.gov/bioproject/PRJNA986898/. The else original contributions presented in the study are included in the article/Supplementary Material. Further inquiries can be directed to the corresponding authors.
