# Supplementary figures and images for: Association between single nucleotide polymorphisms, TGF-β1 promoter methylation, and polycystic ovary syndrome
Source: BMC Pregnancy Childbirth. 2024 Jan 2;24:5. doi: 10.1186/s12884-023-06210-3 (PMC10759533; doi:10.1186/s12884-023-06210-3)

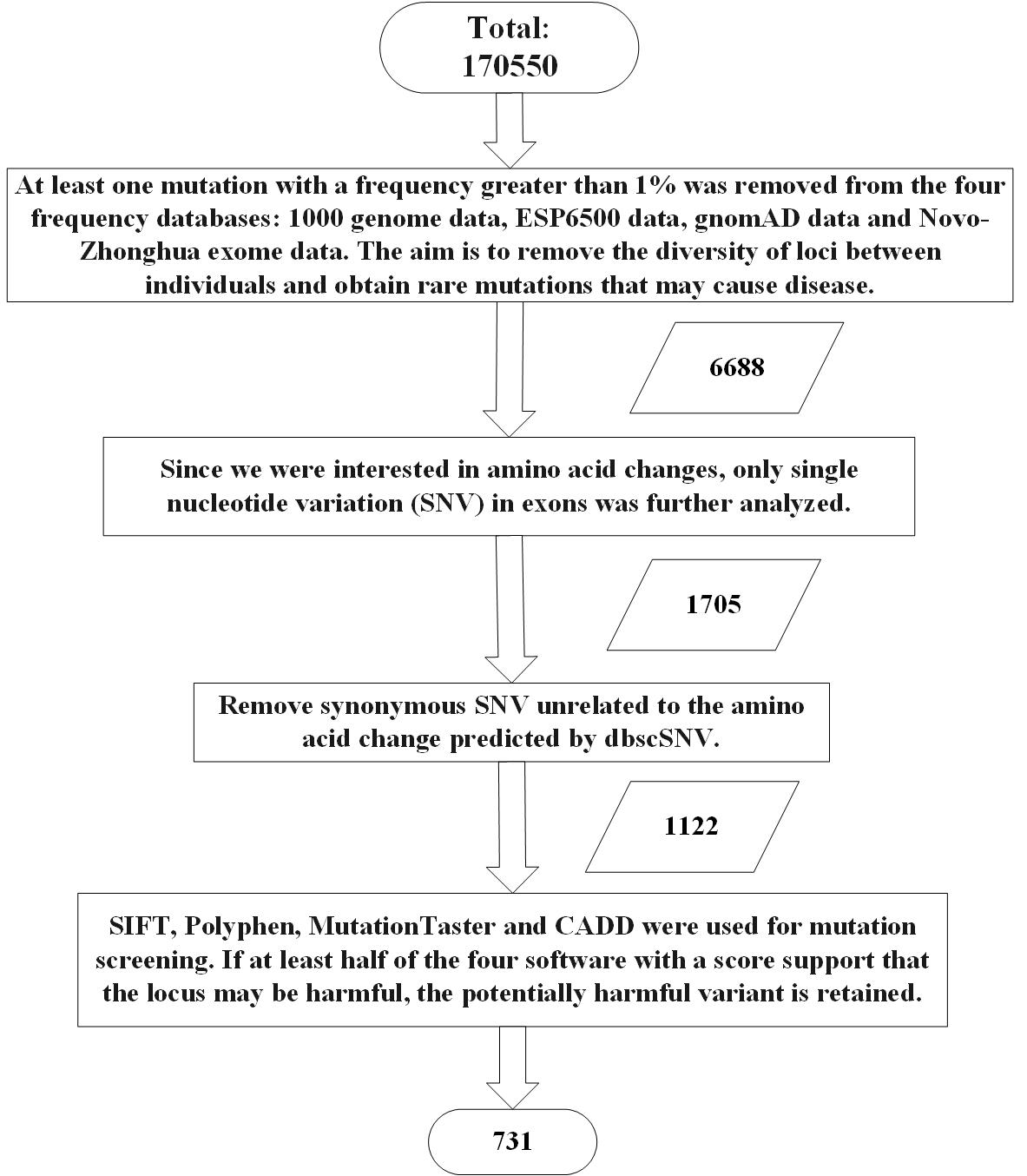
Supplementary Figure 1 : Flow chart of mutation site filtration

Supplement: Supplementary file 1 — Supplementary Material 1 [file 12884_2023_6210_MOESM1_ESM.docx]

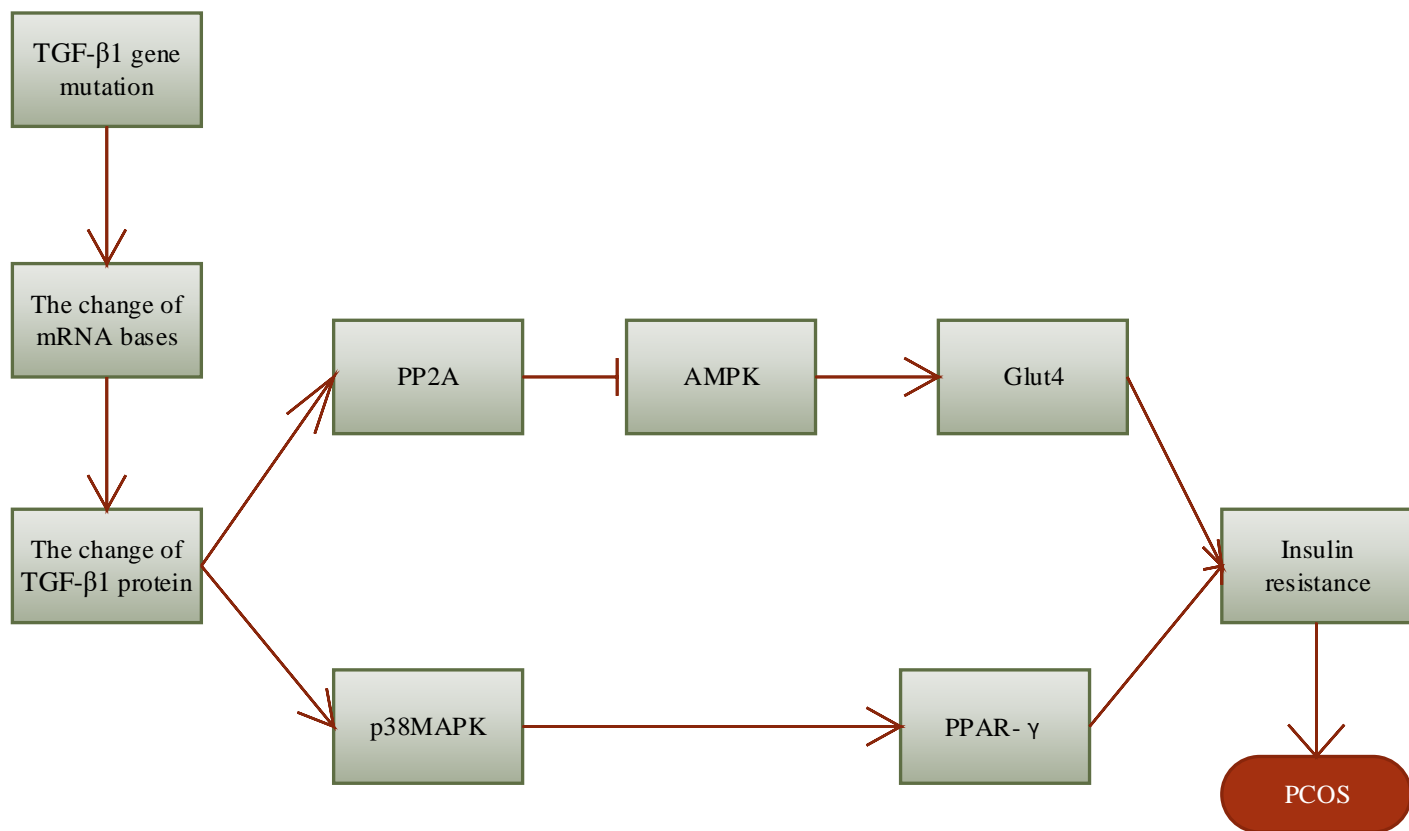

Supplement: Supplementary file 2 — Supplementary Material 2 [file 12884_2023_6210_MOESM2_ESM.pdf]

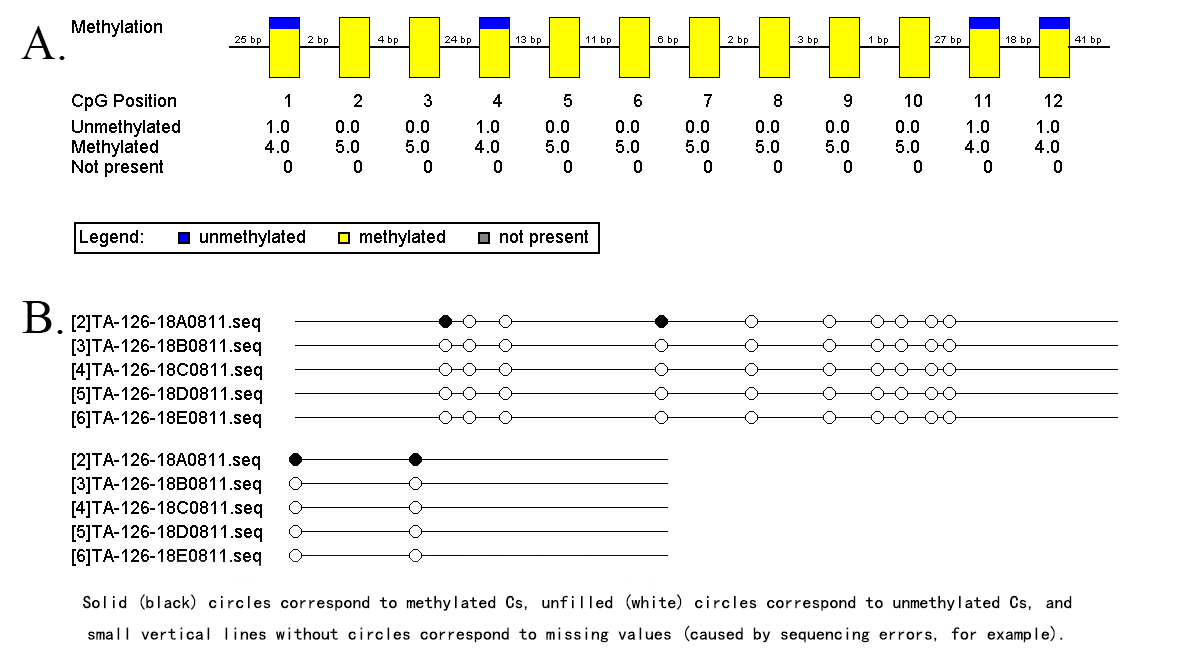

Supplement: Supplementary file 4 — Supplementary Material 4 [file 12884_2023_6210_MOESM4_ESM.png]
